# Discovery-Based Proteomics Identify Skeletal Muscle Mitochondrial Alterations as an Early Metabolic Defect in a Mouse Model of β-Thalassemia

**DOI:** 10.3390/ijms24054402

**Published:** 2023-02-23

**Authors:** Patricia Reboucas, Carine Fillebeen, Amy Botta, Riley Cleverdon, Alexandra P. Steele, Vincent Richard, René P. Zahedi, Christoph H. Borchers, Yan Burelle, Thomas J. Hawke, Kostas Pantopoulos, Gary Sweeney

**Affiliations:** 1 Department of Biology, York University, Toronto, ON M3J 1P3, Canada; 2 Lady Davis Institute for Medical Research, Department of Medicine, McGill University, Montreal, QC H3T 1E2, Canada; 3 Department of Pathology and Molecular Medicine, McMaster University, Hamilton, ON L8S 4L8, Canada; 4 Segal Cancer Proteomics Centre, Lady Davis Institute, Gerald Bronfman Department of Oncology, Jewish General Hospital, McGill University, Montreal, QC H3T 1E2, Canada; 5 Manitoba Centre for Proteomics & Systems Biology, University of Manitoba, Winnipeg, MB R3E 3P4, Canada; 6 Department of Internal Medicine, University of Manitoba, Winnipeg, MB R3T 2N2, Canada; 7 Department of Cellular and Molecular Medicine, Faculty of Medicine, University of Ottawa, Ottawa, ON K1H 8M5, Canada

**Keywords:** thalassemia, iron, skeletal muscle, proteomics, mitochondria

## Abstract

Although metabolic complications are common in thalassemia patients, there is still an unmet need to better understand underlying mechanisms. We used unbiased global proteomics to reveal molecular differences between the th^3/+^ mouse model of thalassemia and wild-type control animals focusing on skeletal muscles at 8 weeks of age. Our data point toward a significantly impaired mitochondrial oxidative phosphorylation. Furthermore, we observed a shift from oxidative fibre types toward more glycolytic fibre types in these animals, which was further supported by larger fibre-type cross-sectional areas in the more oxidative type fibres (type I/type IIa/type IIax hybrid). We also observed an increase in capillary density in th^3/+^ mice, indicative of a compensatory response. Western blotting for mitochondrial oxidative phosphorylation complex proteins and PCR analysis of mitochondrial genes indicated reduced mitochondrial content in the skeletal muscle but not the hearts of th^3/+^ mice. The phenotypic manifestation of these alterations was a small but significant reduction in glucose handling capacity. Overall, this study identified many important alterations in the proteome of th^3/+^ mice, amongst which mitochondrial defects leading to skeletal muscle remodelling and metabolic dysfunction were paramount.

## 1. Introduction

Thalassemia comprises a group of monogenetic disorders that result from the impairment of α- or β-globin expression due to mutations in the α- or β-globin genes [1,2]. The imbalance of α/β-globin chains in the hemoglobin tetramer leads to the formation of hemichromes, reduction in the lifespan of red blood cells and the increased apoptosis of erythroid progenitor cells, which, in turn, triggers extramedullary erythropoiesis and hepatosplenomegaly. The degree of anemia and the overall clinical phenotype vary among patients carrying homozygous or compound heterozygous globin gene mutations. In the most severe form of the disease, known as thalassemia major (TM), frequent blood transfusions are required for sufficient tissue oxygenation and survival [3]. This treatment corrects anemia and attenuates extramedullary erythropoiesis. However, red blood cells contain high amounts of iron (200–250 mg per unit), and, therefore, blood transfusions lead to iron overload. This is further aggravated by increased dietary iron absorption due to the suppression of the iron hormone hepcidin, mainly by erythroferrone and possibly also by additional bone marrow-derived factors, which are induced in response to ineffective erythropoiesis [4]. Indeed, hereditary hemochromatosis is a frequent genetic disorder of iron overload that is caused by the inactivation of hepcidin regulators [5]. 

Large increases in iron levels cannot be fully controlled by iron chelation therapy [6]; therefore, this imbalance can cause cardiomyopathy, diabetes and liver diseases [7]. As many as 50% of TM patients die before the age of 35 [8]. Heart failure, arrhythmia and myocardial infarction are responsible for ~70% of all thalassemic patient deaths [9]. Diabetes, a known driver of cardiovascular disease, also occurs frequently [10,11,12], with one meta-analysis finding the prevalence among TM patients to be 9%, with around 12% having impaired fasting glucose and glucose tolerance [13]. In the early stages of the condition, patients are asymptomatic, and there is often no correlation between serum ferritin levels and the development of cardiac and metabolic dysfunction. Thus, understanding early events that act as drivers of complications in thalassemia is an important research question. Skeletal muscle is the major site of fatty acid catabolism, plays a key role in mediating whole-body glucose homeostasis and is a major determinant of insulin sensitivity. Hence, it is important to consider that impairments to muscle health/metabolism could expedite the progression of complications in persons with thalassemia.

Due to iron overload conditions, the tissues of TM patients experience increased oxidative damage to lipid membranes, proteins and DNA [14]. They have also been shown to exhibit mitochondrial damage, which results in decreased mitochondrial potential [15] and a reduced mitochondrial copy number compared with normal healthy controls [16,17]. Additionally, studies in animal models of thalassemia have demonstrated significant cardiac mitochondrial dysfunction, a contributor to cardiomyopathy [18,19]. Previous studies investigating thalassemia-related alterations in skeletal muscle have documented numerous findings relevant to this work. For example, a study examining muscle biopsies from a small number of α-thalassemia patients indicated a higher capillary tortuosity and unchanged capillary density and diameter [20]. It was concluded that the increased capillary tortuosity would promote oxygen supply to muscle tissues in order to compensate for the lower hemoglobin in those subjects [20]. Ultrastructural changes in the heart of a mouse model and patients with thalassemia included mitochondrial swelling, loss of myofilaments and the presence of lipofuscin, related to the high tissue iron content [21]. Thus, there is increasing evidence that mitochondrial dysfunction is a major manifestation of iron overload in thalassemia, although the temporal and mechanistic aspects of these changes remain to be resolved.

In this study, we used a thalassemic mouse model (th^3/+^) and wild-type control animals at 8 weeks of age. We performed a proteomic analysis of skeletal muscle to discover differentially expressed proteins. Data input into an Ingenuity pathway analysis allowed for the identification of key pathways, which differed between genotypes. String analysis was used to identify major alterations in protein interactomes. Key data were further verified via standard histological, Western blotting and qPCR approaches. The impact on metabolic genotype was determined using glucose and insulin tolerance tests.

## 2. Results

### 2.1. Hematological and Iron Phenotype of 8-Week-Old th^3/+^ Mice

**Figure 1:**
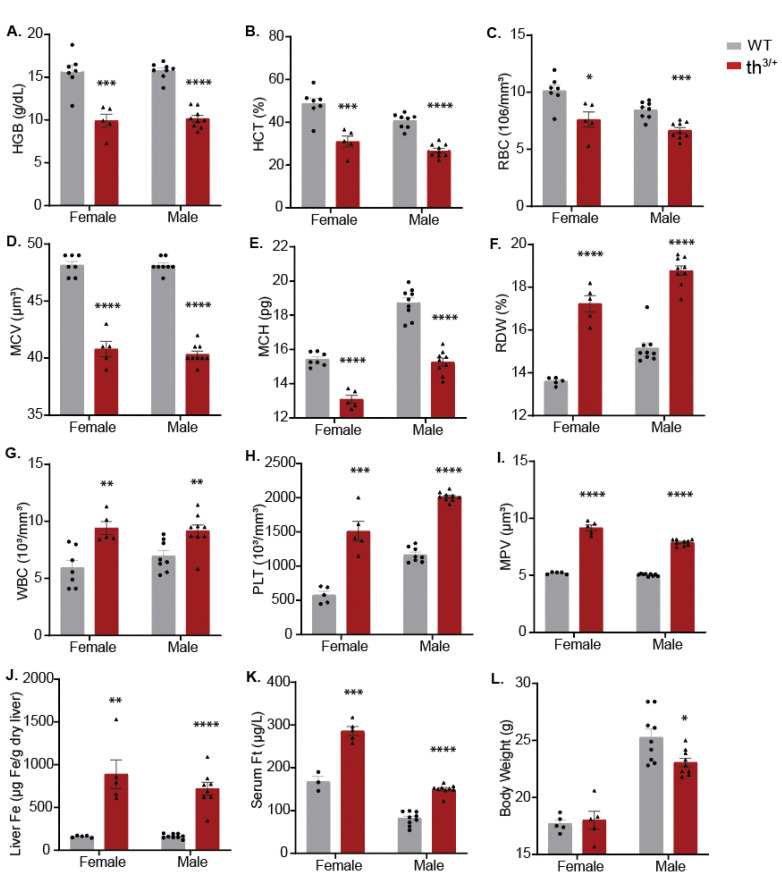
Hematological and iron phenotypes of 8-week-old th^3/+^ mice. Male and female animals (*n* = 3 wt and *n* = 5 th^3/+^) were euthanized, and blood was collected for hematological analysis. Serum was prepared for ferritin assay, and livers were harvested for iron quantification. (**A**) Hemoglobin (HGB); (**B**) hematocrit (HCT); (**C**) red blood cells (RBC); (**D**) mean corpuscular volume (MCV); (**E**) mean corpuscular hemoglobin (MCH); (**F**) red cell distribution width (RDW); (**G**) white blood cells (WBC); (**H**) platelet (PLT) count; (**I**) mean platelet volume (MPV); (**J**) liver iron content; (**K**) serum ferritin; (**L**) body weight. Data are represented as mean ± SEM. Statistically significant differences across genotypes are indicated: * *p* < 0.05; ** *p* < 0.01; *** *p* < 0.001; **** *p* < 0.0001.

Hematological analysis validated the thalassemia phenotype of th^3/+^ mice at the age of 8 weeks. When compared with the wild-type controls, the th^3/+^ animals exhibited reduced hemoglobin (HGB) content (Figure 1A), hematocrit (HCT) (Figure 1B), red blood cell (RBC) count (Figure 1C), mean corpuscular volume (MCV) (Figure 1D) and mean corpuscular hemoglobin (MCH) (Figure 1E). Additionally, they manifested increased red cell distribution width (RDW) (Figure 1F), white blood cell (WBC) count (Figure 1G), platelet (PLT) count (Figure 1H) and mean platelet volume (MPV) (Figure 1I). This typical hematological phenotype of thalassemia in th^3/+^ mice was accompanied by systemic iron overload, as indicated by the increased liver iron content (LIC) (Figure 1J) and serum ferritin (Figure 1K), a reflection of LIC. There was no genotype-specific difference in the body weight of the female animals, which was lower compared with that of the males; however, the body weight of the th^3/+^ males was significantly reduced vs. the wild-type (Figure 1L).

### 2.2. Proteomics Analysis of Skeletal Muscle

**Figure 2:**
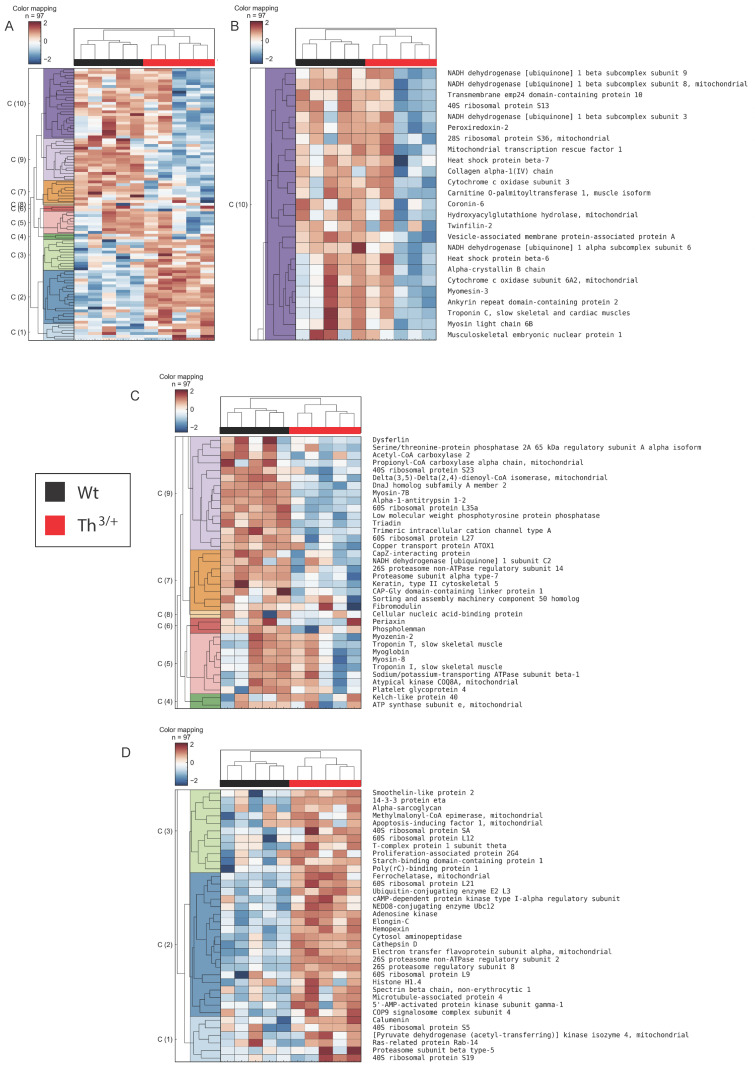
Remodelling of skeletal muscle proteome in 8-week-old th^3/+^ mice. (**A**–**D**) Unsupervised hierarchical clustering (Euclidean distance, complete) was performed using label-free protein quantitation data from all proteins showing an adjusted *p*-value of <0.05 and a fold change cut-off exceeding twofold in comparison with the WT and th^3/+^ groups. Normalized protein abundances underwent log2-transformation and z-score normalization prior to clustering. Figure (**B**–**D**) show individual clusters as well as protein annotation for 10 clusters, 4–9 (showing overall decreases) and 1–3 (showing overall increases), respectively.

In order to investigate potential alterations to the skeletal muscle proteome of th^3/+^ mice, we conducted an unbiased quantitative mass spectrometry-based proteomics study using label-free quantitation. We measured differences in protein expression relative to control mice. Using this approach, we were able to quantify 891 proteins with a minimum of 1 protein-group-unique peptide (80% having ≥2 protein-unique peptides). Of these quantified proteins, 97 showed statistically significant differential expression based on an FDR-adjusted *p*-value of <0.05 and a fold change cut-off of twofold. Based on these proteins, we performed hierarchical clustering, which revealed several clusters of regulated networks (Figure 2A). Clusters 4–10, generally representing proteins with decreased expression in the thalassemic group vs. controls (Figure 2B,C), and clusters 1–3, representing proteins with generally increased expression (Figure 2D).

**Figure 3:**
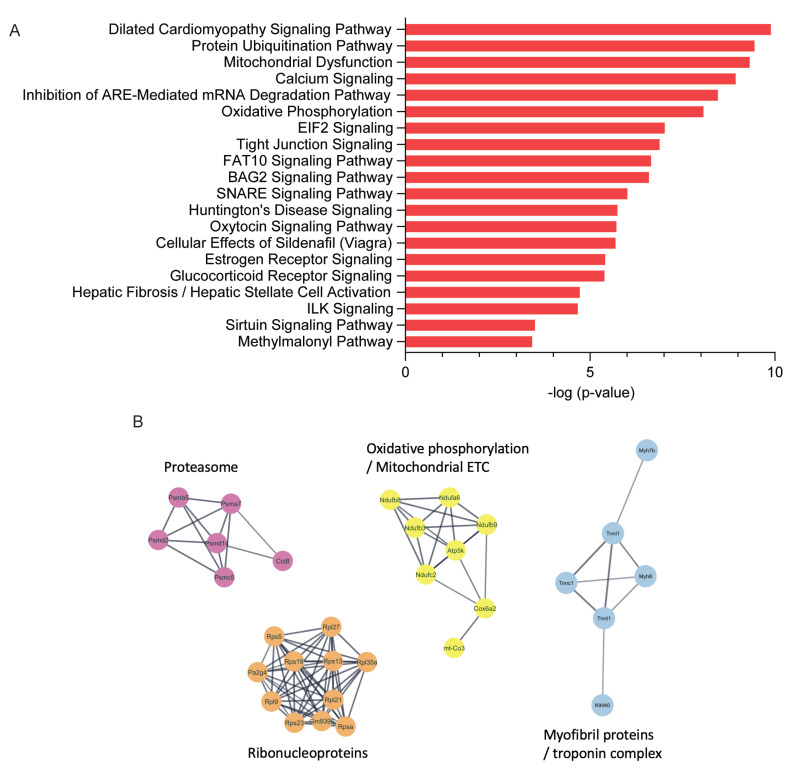
Ingenuity pathway analysis (IPA) and functional enrichment of protein–protein interaction networks. (**A**) Canonical pathway enrichment analysis generated using Qiagen IPA software based on all differentially expressed proteins. (**B**) STRING networks based on all differentially expressed proteins show enrichment in particular GO-terms and KEGG pathways, including proteasomal proteins, proteins involved in OXPHOS and the mitochondrial electron transport chain (ETC), as well as ribonucleoproteins and myofibril/troponin complex components.

Ingenuity pathway analysis (IPA) demonstrated functional enrichment for components involved in cardiomyopathy signalling, protein ubiquitination and mitochondrial dysfunction, among others (Figure 3A). The further functional enrichment of protein–protein interaction networks using StringDB indicated that regulated components were enriched for members of the proteasome complex, ribonucleoproteins, mitochondrial proteins involved in oxidative phosphorylation and electron transport, as well as myofibril proteins and components of the troponin complex (Figure 3B).

### 2.3. Partial Deletion of β-Globin Gene Results in Altered Phenotype in the Gastrocnemius Muscle of Thalassemia Mouse Model

**Figure 4:**
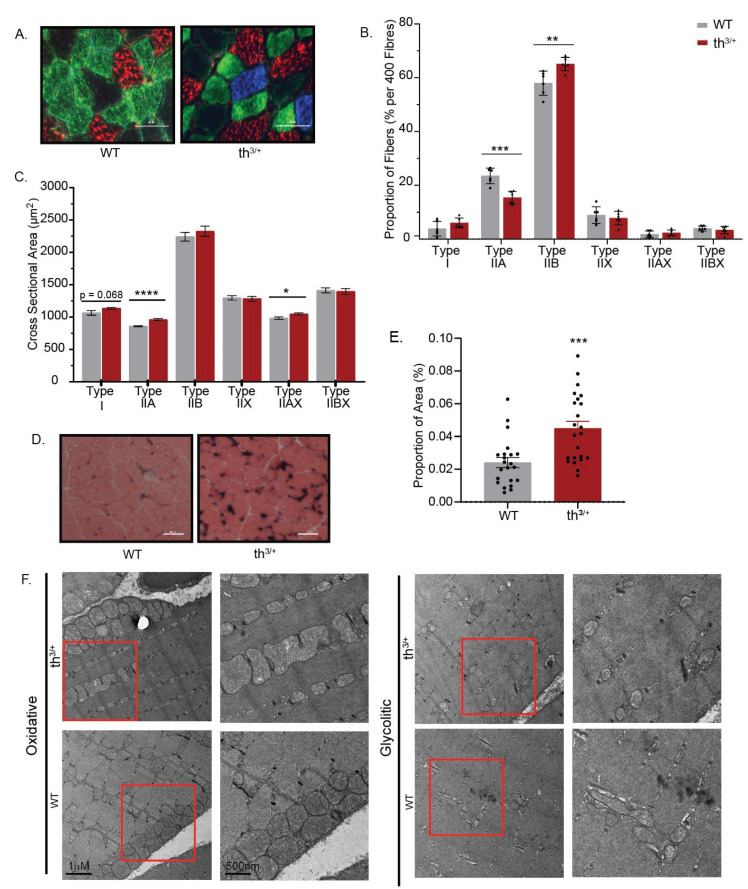
Partial deletion of β-globin gene results in altered phenotype in the gastrocnemius muscle. (**A**) Representative images of th^3/+^ and WT gastrocnemius-fibre-type staining. each colour represents a different fiber type: blue is Type I, green is type IIA, red is Type IIB, blue is type I and black is Type IIX. The scale bar represents 60 μm. (**B**) Immunohistochemical analysis of fibre type proportions per 400 fibres in the gastrocnemius of thalassemia (th^3/+^) and wild-type (WT) mice (N = 4 with 100 fibres quantified per muscle). (**C**) Cross-sectional area (μm^2^) of fibre types in the gastrocnemius. (**D**) Representative images of th^3/+^ and WT gastrocnemius alkaline phosphatase stain. Capillaries are indicated by dark colour, with background of muscle fibers as red. The scale bar represents 50 μm. (**E**) Percentage of alkaline phosphatase stain present in selected regions of interest in th^3/+^ and WT gastrocnemius muscle. (**F**) Representative TEM images for both oxidative and glycolytic muscle samples, with a red-highlighted box shown at higher magnification on right. *n* = 6 in both th^3/+^ and WT groups. Data in graphs are mean ± SEM. Statistical differences from independent *t*-test analysis (* *p <* 0.05, ** p < 0.01, *** p < 0.001, **** *p* < 0.0001) versus WT.

Gastrocnemius muscles from th^3/+^ mice had a significantly lower proportion of type IIA fibres vs. WT (*p* = 0.0003) and a significant increase in the proportion of type IIB fibres vs. WT (*p* = 0.0066) (Figure 4A,B). In gastrocnemius muscles, cross-sectional area (CSA) differences between fibre types were present. A trend toward a greater CSA for type I fibres existed in th^3/+^ compared with the WT mice (*p* = 0.68). The CSA of the type IIa fibres was significantly greater in th^3/+^ compared with WT mice (*p* < 0.0001). The CSA of the type II a/x hybrid fibres was also significantly greater in th^3/+^ vs. WT mice (*p* = 0.021) (Figure 4C). To observe the influence of the thalassemia phenotype on muscle capillarization, we investigated the proportion of capillaries in regions of interest with an alkaline phosphatase stain. There was an increase in capillary density in gastrocnemius muscles from th^3/+^ mice shown by a significant increase in alkaline phosphatase-stained areas compared with WT mice (*p* = 0.0003) (Figure 4D,E). Figure 4F shows representative images of oxidative and glycolytic muscle samples (the red-highlighted area shown at higher magnification), although no overt differences were observed between genotypes.

### 2.4. Mitochondrial Content Is Changed in Skeletal Muscle of Thalassemia Model

**Figure 5:**
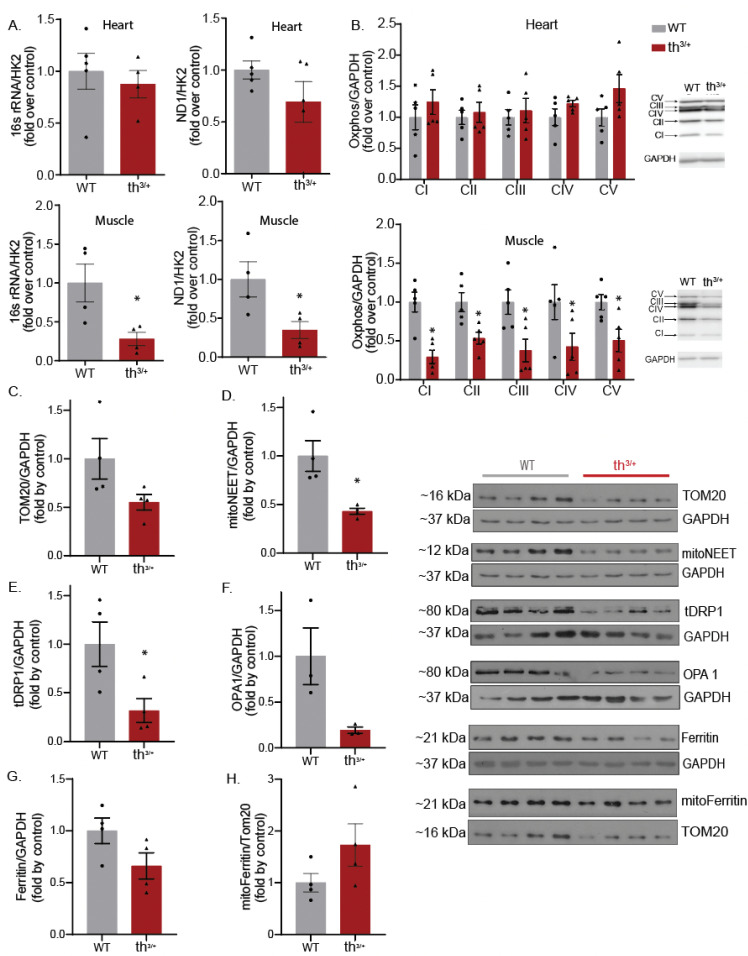
Mitochondrial content change in skeletal muscle of thalassemia model. Relative quantification was performed on wild-type and th^3/+^ heart or skeletal muscle samples (**A**) using qPCR via the amplification of 16S and ND1 genes representing the stable part of mtDNA and normalized against hexokinase gene 2 (HK2). Representative Western blots for OXPHOS (**B**), from heart or skeletal muscle tissue (*n* = 5), with quantitation shown in the graph for complex I-V. Representative Western blots for (**C**) TOM20, *n* = 4; (**D**) mitoNEET, *n* = 4; (**E**) tDRP1, *n* = 4; (**F**) OPA1, *n* = 3; (**G**) Ferritin, *n* = 4; (**H**) mitoFerritin, *n* = 4 in both th^3/+^ and WT groups, with quantitative analysis shown in graphs as mean ± SEM. Statistical differences were calculated using Student’s *t*-test (* *p <* 0.05) versus WT.

Mitochondrial DNA (mtDNA) quantification has been used as a reliable indicator of mitochondrial quantity, as mtDNA levels remain almost constant in healthy organisms. Analysis of the 8-week-old tissue data showed significantly reduced mitochondrial content in the skeletal muscle of the th^3/+^ mice compared with the controls (Figure 5A). Skeletal muscle from the th^3/+^ mice had significantly decreased content for mitochondrial markers, 16S rRNA and ND1, whereas, in heart tissue, there was no significant difference in mtDNA markers (Figure 5A). Similarly, when we examined the content of OXPHOS complex I-V, there was a significant decrease in the skeletal muscle, but not the hearts, from th^3/+^ mice (Figure 5B). In addition, we observed a decrease in the expression of the mitochondrial proteins TOM20, MitoNEET, DRP1 and OPA1 (Figure 5C–F) in skeletal muscle from th^3/+^ mice. Interestingly, there was no significant difference in ferritin or mitoFerritin expression between genotypes when adjusted to the GAPDH and TOM20 controls, respectively (Figure 5G,H).

### 2.5. Metabolic Analysis of 8-Week-Old th^3/+^ Mice

**Figure 6:**
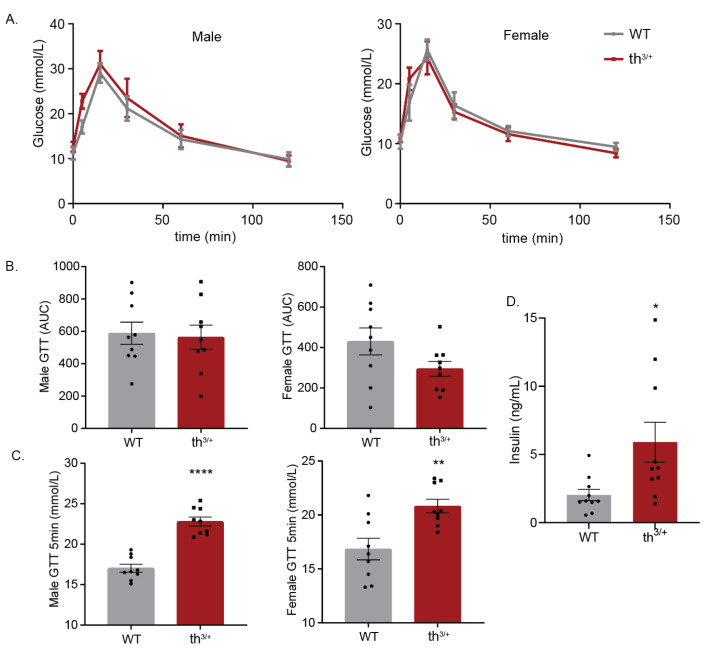
Metabolic analysis of 8-week-old th^3/+^ mice. Male and female wild-type mice and th^3/+^ littermates were subjected to GTT. (**A**) Intraperitoneal glucose tolerance test (IpGTT) after overnight fasting in male and female WT and th^3/+^ mice. (**B**) Area under curve (AUC) of IpGTT in male and female mice from 6A. (**C**) Quantification of the first 5 min of the IpGTT in males and females from 6A. (**D**) Serum insulin levels from fasted male WT and th^3/+^ mice. *n* = 10 in both th^3/+^ and WT groups. Data in graphs are mean ± SEM. Statistical differences were calculated using two-way ANOVA analysis. Unpaired *t-test* analysis was performed for Figure 5A–D. * p < 0.05,  ** p < 0.01, **** p < 0.0001 versus WT.

To determine how these changes in skeletal muscle are linked to diabetes, a glucose tolerance test (GTT) was performed. No significant difference between wild-type and thalassemia mice was observed regardless of sex (Figure 6A or B). A significant increase in blood glucose was, however, observed during the first 5 min of the GTT in both male and female th^3/+^ mice (Figure 6C). There was a higher insulin level (indicative of reduced insulin sensitivity) in 8-week-old th^3+/−^ mice at the first 5 min post-glucose injection timepoint (Figure 6D).

## 3. Discussion

Studies with thalassemia patients have demonstrated an increased risk of diabetes, heart disease and metabolic syndrome [22,23]. Interestingly, approximately 30% of metabolic syndrome patients exhibit iron overload, and the term dysmetabolic iron overload syndrome (DIOS) has been coined to describe this population [24]. Interventions to reduce iron excess, such as via venesection or the use of chelators, have been shown to improve insulin sensitivity and delay the onset of type 2 diabetes and heart failure [25,26], although this approach has not always been successful [27]. Based on the overall knowledge derived from studies to date, we postulated that iron overload itself may be a primary driver of impairments in the metabolic health of skeletal muscle. This work is of clinical significance, as additional insight into mechanisms underlying the development of metabolic complications in thalassemia is needed, particularly with a view to early intervention. Thus, we used an established mouse model of thalassemia and a proteomics-driven approach, which directed our analyses to mitochondrial alterations in skeletal muscle.

Here, we show that the thalassemia phenotype is associated with the substantial remodelling of the skeletal muscle proteome. Based upon our standard analytical criteria, in this study, there were 97 quantified proteins that showed statistically significant differential expression. Hierarchical clustering revealed several clusters of regulated networks. Broadly speaking, the most prominent changes were observed in proteins related to mitochondrial pathways and protein ubiquitination. Accordingly, additional functional enrichment of protein–protein interaction networks using StringDB indicated that regulated components were enriched for members of the proteasome complex and mitochondrial proteins involved in oxidative phosphorylation and electron transport. In this study, we focused mainly on further investigating the proteomics signature for mitochondrial dysfunction. The association between mitochondrial defects and thalassemia has been indicated by various previous studies [28,29]. In erythroblasts from thalassemia patients versus controls, decreased mitochondrial oxidative phosphorylation was observed [17]. Impaired fatty acid oxidation in mitochondria was suggested to be related to decreased carnitine levels found in the circulation of individuals with thalassemia [30]. A study using reticulocytes showed the increased expression of mitochondrial ferritin in patients with α-thalassemia, suggesting that iron excess in mitochondria occurs, and this may be important in mitochondrial dysfunction [31]. There may be many contributors to skeletal muscle mitochondrial remodelling in thalassemia, one of which may be the direct effects of labile iron excess, in particular, intramitochondrial iron overload [28]. This is known to attenuate mitochondrial respiration by causing a decrease in cytochrome C oxidase [32], a finding we observed here in our study. Interestingly, targeting improved cytochrome c oxidase content via in vitro-transcribed (IVT)-mRNA delivery has been proposed as a therapeutic approach applicable to thalassemia [33]. In addition to mitochondrial metabolic dysfunction, our data also indicated potential defects in antioxidative mechanisms, thus exacerbating the impact of mitochondrial damage. For example, predominant in our dataset was decreased NADPH dehydrogenase content. It has been suggested that the peroxiredoxin-2-mediated induction of NADPH dehydrogenase quinone-1 is an important adaptive response to counteract oxidative stress [34], and it is likely that lack of this in the muscle of th^3/+^ mice correlates with the development of metabolic dysfunction. It should be noted that, while the proteomics analysis presented here indicates many interesting findings, one potential limitation is that the number of animals used is relatively small.

One of the primary drivers for the changes in proteome observed here may be the need for skeletal muscles to adapt to reduced (due to anemia) oxygen delivery. Indeed, the idea that anemia may be modifying the morphology of skeletal muscle is consistent with the present results (increased capillary density) and previous work showing an increased capillary tortuosity in the muscles of thalassemia patients, a phenomenon the authors speculated would promote an increased oxygen supply to muscle tissue [20]. In this study, we observed a shift away from oxidative and mitochondrial-dependent fibre types toward more glycolytic and less mitochondrial-dependent fibre types. This response is consistent with the compensation of reduced oxygen delivery to skeletal muscles with glycolytic metabolism, yet it comes at the cost of inefficiency. This increase in the glycolytic phenotype of the muscle is also in agreeance with a reduction in the mitochondrial capacity in thalassemia mice, which we observed based on the proteomics profile; reduced mitochondrial DNA markers and Western blotting showing reduced OXPHOS expression. Based on glucose tolerance tests, we observed a mild insulin resistance phenotype in th^3/+^ mice at 8 weeks of age. The development of insulin resistance in muscle is expected to occur following mitochondrial dysfunction [35] or iron overload [36,37]. It is likely that as the mice age this defect would become more prominent [38].

Another aspect of the proteomic signature we found in this study that is likely of great significance in the context of the related literature is altered protein homeostasis pathways, both proteasome- and lysosome-mediated. It is well established that the ubiquitin–proteasome system and autophagy both play an important role in thalassemia via the excess amounts of free α-globin being processed via these protein quality control mechanisms [39,40]. Elevated proteasome activity was found in a previous study using red blood cell units from fourteen β-thalassemia donors versus sex- and aged-matched controls [41]. A study of the platelet proteome of X-linked thrombocytopenia with thalassemia patient pathway analysis revealed protein ubiquitination as a principal alteration [42]. Many and varied connections of autophagy with thalassemia have been reported, particularly the regulation of β-thalassemia erythropoiesis [43]. However, it is also likely that altered autophagy at the tissue level may have an important role in disease pathogenesis. Specifically, altered skeletal muscle autophagy, particularly mitophagy, can certainly contribute to muscle mitochondrial damage and metabolic dysfunction [44]. Excess labile iron is also likely to be a direct driver of the changes seen here, as persistent high levels of iron attenuate skeletal muscle autophagy by inhibiting autophagosome lysosome regeneration (ALR) [36]. Muscle can normally initiate endogenous mechanisms to protect itself from slightly elevated iron levels, and autophagy is a critical part of this response [45]. However, our data indicated this may not occur in th^3/+^ mice muscle, rendering them more susceptible to cellular damage. Excess iron has also been shown to attenuate the ubiquitin–proteasome protein quality control system in various cell types [46,47].

As far as we are aware, this is the first study of the skeletal muscle proteome in either human patients or a mouse model of thalassemia. Nevertheless, other proteomics-based studies have been conducted [48]. Analysis of the plasma proteome of patients with β-thalassemia versus healthy controls identified 13 potential biomarkers [49]. Interestingly global correlation analysis identified some pathways that can be cross-referenced with findings from our study; for instance hypertrophic/dilated cardiomyopathy signature being prominent and altered lysosomal function, especially cathepsins. In keeping with a cardiomyopathy signature, serum lipidomics analysis indicated that transfusion-dependent thalassemia patients had elevated triacylglycerols and long-chain acylcarnitines, with lower ether phospholipids or plasmalogens, sphingomyelins and cholesterol esters, reminiscent of what has been previously characterized in cardiometabolic diseases [5]. Untargeted metabolomics studies have also begun to characterize the metabolic defects in thalassemic individuals and how this can be altered upon intervention with hydroxyurea [50,51]. In summary, various types of omics-driven studies have established defective metabolic pathways in thalassemia that must now be further investigated. The most appropriate targets need to be defined and interventions tested.

Overall, in 8-week-old th^3/+^ mice, we identified alterations in the proteome that point toward early mitochondrial alterations and dysfunction. A lack of hemoglobin-mediated oxygen delivery to muscle, in concert with this reduced mitochondrial capacity, correlated with a switch toward glycolytic muscle fibres and an increase in capillarization. These remodelling events are logical in terms of maximizing the use of available oxygen. Thus, we identified many important alterations in the proteome of th^3/+^ mice, among which, mitochondrial defects associated with skeletal muscle remodelling and metabolic dysfunction were paramount. From this and various other proteomics studies related to β-thalassemia, an important insight into disease pathogenesis is beginning to emerge, with mitochondrial function and protein degradation (lysosome and proteasome) being two prominent cellular processes that are perturbed.

## 4. Materials and Methods

### 4.1. Experimental Animals, Serum and Tissue Collection

Eight-week-old mice heterozygous for the β-globin gene deletion (th^3/+^; also known as Hbb^th3/+^) on C57BL/6 background were used as a model of thalassemia and compared with wild-type littermates [52]. This mouse model is most akin to human thalassemia intermedia, as these mice do not require blood transfusions. The animals, both male and female, were housed in macrolone cages (up to 5 mice/cage, 12:12 h light–dark cycle: 7 am–7 pm; 22 ± 1 °C, 60 ± 5% humidity) and were allowed ad libitum access to chow and drinking water. Experimental procedures were approved by the Animal Care Committee of McGill University (protocol 4966). At the endpoint, blood was collected via cardiac puncture following anesthesia under isoflurane; tissues were then rapidly collected and snap-frozen in liquid nitrogen followed by storage at −80 °C until further use.

### 4.2. Hematological Analysis, Serum Biochemistry and Quantification of Liver Iron

Hematological parameters were determined with the Scil Vet-ABC hematology analyzer. Serum was prepared by using micro-Z-gel tubes with a clotting activator (Sarstedt) and kept frozen at −20 °C until analysis. Serum ferritin was determined at the Biochemistry Department of the Montreal Jewish General Hospital using a Roche Hitachi 917 Chemistry Analyzer. Tissue iron was quantified with a ferrozine assay, as previously described [53].

### 4.3. Global Proteome Analysis of WT and Thalassemic Mouse Muscle Tissues

Gastrocnemius muscle tissue samples from wild-type and th^3/+^ mice (total, *n* = 10) were lysed in buffer containing 5% sodium dodecyl sulphate (SDS) and 100 mM TRIS pH 7.8. Samples were subsequently heated to 99 °C for 10 min and subjected to probe-based sonication using a Thermo Sonic Dismembrator at 25% amplitude for 3 cycles × 5 s. Remaining debris was pelleted by centrifugation at 20,000× *g* for 5 min. An aliquot of the supernatant was diluted to <1% SDS and used for estimation of protein concentration via bicinchoninic acid assay (BCA) (Pierce, cat# 23225). Lysates were clarified by centrifugation at 14,000× *g* for 5 min and transferred to a new reaction tube; disulphide bonds were reduced by the addition of tris(2-carboxyethyl)phosphine (TCEP) to a final concentration of 20 mM and incubated at 60 °C for 30 min. Free cysteines were alkylated using iodoacetamide at a final concentration of 30 mM and subsequent incubation at 37 °C for 30 min in the dark. An equivalent of 200 µg of total protein was used for proteolytic digestion via suspension trapping (S-TRAP) [54]. Proteins were acidified by adding phosphoric acid to a final concentration of 1.3% *v*/*v*. Samples were then diluted 6-fold in STRAP loading buffer (9:1 methanol:water in 100 mM TRIS, pH 7.8) and loaded onto an S-TRAP Mini cartridge (Protifi LLC, Huntington NY) prior to centrifugation at 2000× *g* for 2 min. Samples were washed three times with 350 µL of STRAP loading buffer and proteolytically digested using trypsin (Sigma, Toronto, Canada) at a 1:10 enzyme-to-substrate ratio for 16 h at 37 °C. Peptides were sequentially eluted in 50 mM ammonium bicarbonate, 0.1% formic acid in water and 50% acetonitrile. Peptide-containing samples underwent solid phase extraction using Oasis HLB, 30 mg, 1CC cartridges (Waters). Peptide samples were dried and reconstituted in 0.1% trifluoro acetic acid (TFA) prior to analysis using mass spectrometry.

### 4.4. LC-MS/MS Acquisition and Data Analysis

Samples were analyzed via data-dependent acquisition (DDA) using an Easy-nLC 1200 online coupled to a Q Exactive Plus (both Thermo Fisher Scientific). Samples were first loaded onto a pre-column (Acclaim PepMap 100 C18, 3 µm particle size, 75 µm inner diameter × 2 cm length) in 0.1% formic acid (buffer A). Peptides were then separated using a 100 min binary gradient ranging from 3–40% B (84% acetonitrile, 0.1% formic acid) on the analytical column (Acclaim PepMap 100 C18, 2 µm particle size, 75 µm inner diameter × 25 cm length) at 300 nL/min. MS spectra were acquired from *m*/*z* 350–1500 at a resolution of 70,000, with an automatic gain control (AGC) target of 1 × 10^6^ ions and a maximum injection time of 50 ms. The 15 most intense ions (charge states +2 to +4) were isolated with a window of *m*/*z* 1.2, an AGC target of 2 × 10^4^ and a maximum injection time of 64 ms and fragmented using a normalized higher-energy collisional dissociation (HCD) energy of 28. MS/MS spectra were acquired at a resolution of 17,500, and the dynamic exclusion was set to 40 s. DDA MS raw data were processed with Proteome Discoverer 2.5 (Thermo Scientific) and searched using Sequest HT against the canonical mouse SwissProt FASTA database downloaded from UniProt. The enzyme specificity was set to trypsin with a maximum of 2 missed cleavages. Carbamidomethylation of cysteine was set as the static modification and methionine oxidation as the variable modification. The precursor ion mass tolerance was set to 10 ppm, and the product ion mass tolerance was set to 0.02 Da. The percolator node was used, and the data were filtered using a false discovery rate (FDR) cut-off of 1% at both the peptide and protein level. The Minora feature detector node of Proteome Discoverer was used for precursor-based label-free quantitation.

### 4.5. Analysis of Proteomics Data

Proteins quantified by at least one protein-unique peptide were further filtered based on a minimum of >50% valid values in at least one of the two sample groups. Remaining missing values were imputed by low abundance sampling within Proteome Discoverer 2.5. LFQ abundances were scaled (normalized) based on the total amount of quantified peptides, and abundance ratios were calculated as the ratio of grouped protein abundances. Statistical significance was determined using background-adjusted t-tests and adjusted for false discovery rate (FDR) using the Benjamini–Hochberg method within Proteome Discoverer 2.5. Regulation was defined on the basis of having an adjusted *p*-value of less than 0.05 and an expression ratio cut-off of 2-fold. Dimensional reduction via principal component analysis and hierarchical clustering was conducted using the normalized protein and phosphopeptide LFQ abundances in Instant Clue (http://www.instantclue.uni-koeln.de/). Functional enrichment analysis of protein–protein interaction networks was performed using STRINGDB analysis [55] with the StringApp for visualization within Cytoscape (v 3.9.1) [56].

### 4.6. Fibre Typing

Muscle fibre typing was performed according to the protocol by Bloemberg and Quadrilatero [57]. Briefly, 10 μm of gastrocnemius muscle sections were cut with the Leica CM1850 Cryostat, maintained at −20 °C. The muscle sections were blocked with 10% goat serum (Vector Laboratories, S-1000) for 60 min followed by incubation with a primary antibody cocktail (Developmental Springs Hybridoma Bank, Iowa City, IO; BA-F8 1:50, SC-71 1:600, BF-F3 1:100) against three myosin heavy chain (MHC) isoforms (type I, type IIA and type IIB) for 120 min. Sections were washed with PBS and then incubated with the appropriate secondary antibodies (Alexa Fluor 350 IgG2b 1:500, Alexa Fluor 488 IgG1 1:500, Alexa Fluor 555 IgM 1:500) for 60 min. 

### 4.7. Alkaline Phosphatase Stain

Gastrocnemius (10 μm) sections were cut and stained for alkaline phosphatase with SIGMAFAST (BCIP/NBT (B5655-25TAB)) dissolved in 10 mL of dH₂O to determine capillary density. Muscle sections were incubated with the alkaline phosphatase solution for 15 min at 37 °C, whereafter, they were rinsed with dH₂O and counterstained with eosin (0.5% *v*/*w* in dH₂O).

### 4.8. Transmission Electron Microscopy (TEM)

Gastrocnemius muscle was dissected in 1 mm^3^ for analysis via TEM. After two washes with ice-cold 0.2 M sodium cacodylate buffer containing 0.1% calcium chloride, pH 7.4, samples were fixed overnight at 4 °C in 2.5% glutaraldehyde and washed 3× with washing buffer. Pellets were post-fixed with 1% aqueous OsO_4_ + 1.5% aqueous potassium ferrocyanide for 1 h and washed 3× with washing buffer. Specimens were dehydrated in a graded alcohol series, infiltrated with graded epon:alcohol and embedded in epon. Sections were polymerized at 58 °C for 48 h. Ultrathin sections (90–100 nm thick) were prepared with a diamond knife using a Reichert Ultracut E-ultramicrotome, placed on 200 mesh copper grids and stained with 2% uranyl acetate for 6 min and Reynold’s lead for 5 min. Grids were then examined with transmission electron microscopy.

### 4.9. PCR Analysis

Samples were prepared and processed following the methods and protocol, as described before [58]. The primers used were 16S rRNA: Forward: 5′-CCGCAAGGGAAAGATGAAAGAC-3′ Reverse: 5′-TCGTTTGGTTTCGGGGTTTC-3′, ND1: Forward: 5′-CTAGCAGAAACAAACCGGGC-3′ Reverse: 5′-CCGGCTGCGTATTCTACGTT-3′, HK2: Forward: 5′-GCCAGCCTCTCCTGATTTTAGTGT-3′ Reverse: 5′-GGGAACACAAAAGACCTCTTCTGG-3′.

### 4.10. Western Blotting

Skeletal muscle, gastrocnemius and heart tissues were chop-frozen or pulverized with mortar and pestle in liquid nitrogen. The powdered tissue was then suspended in lysis buffer and homogenized with beads and then incubated on a rotating rocker for 1 h at 4 °C, followed by centrifuging samples at 1000 rpm for 10 min at 4 °C. The supernatant was transferred to a new Eppendorf tube, concentration was measured using BCA and samples were kept at −80 °C. Samples were then centrifuged at 10,000 rpm for 5 min at 4 °C and denatured at 95 °C for 5 min. Samples were run on 8%, 12% and 15% SDS-PAGE gels conducted at approximately 90 V for 2 h, followed by transfer to a polyvinylidene difluoride (PVDF) membrane at 120 V for 1.5 h. Membranes were blocked in 3% bovine serum albumin (BSA) blocking solution for 1 h at room temperature, followed by incubation in a 1:1000 dilution of the primary antibody overnight. The next day, membranes were washed and incubated with a secondary antibody in 1:5000 dilution for 1 h at room temperature. Membranes were activated using Clarity Western ECL Substrate solution and visualized using X-ray film development techniques. Western blot (WB) band intensity was quantified using ImageJ software and normalized to specific loading control. The following primary antibodies were used in this study: TOM20 (Cat#42406), CISD1/mitoNEET (Cat#83775), total DRP1 (Cat#8570), OPA1 (Cat#80471), GAPDH (Cat#2118). They were purchased from Cell Signalling Technology, Beverley, MA. Ferritin (Cat#PA1-29381) was purchased from Invitrogen, Toronto, Canada. Mitochondrial ferritin (Cat#ab66111) and total OXPHOS (Cat#ab110413) were purchased from Abcam. The following secondary antibodies were used: anti-rabbit immunoglobulin G horseradish peroxidase-linked antibody (Cat#7074) and anti-mouse immunoglobulin G horseradish peroxidase-linked antibody (Cat#7076), from Cell Signalling Technology.

### 4.11. Glucose Tolerance Test (GTT) and Analysis of Insulin

Wild-type and th^3/+^ mice littermates fasted for 5 h before the experiment. Subsequently, the animals were injected with 100 g/kg glucose ip. Blood glucose levels were measured by using the OneTouch Verio Flex glucose meter.

### 4.12. Statistical Analysis

Data are presented as mean ±SEM. Statistical significance between treatment groups was calculated using an unpaired Student’s *t*-test when comparing two groups. One-way ANOVA and two-way ANOVA were used for the comparison of more than two groups. A *p*-value of <0.05 was considered statistically significant, and it was plotted with GraphPad Prism 9.

## Data Availability

Original data are available from the authors upon reasonable request.
